# Patients Age 40 Years and Younger With Multiple Myeloma Have the Same Prognosis as Older Patients: An Analysis of Real-World Patients' Evidence From Latin America

**DOI:** 10.1200/GO.23.00182

**Published:** 2023-12-07

**Authors:** Humberto Martínez-Cordero, Camila Peña, Natalia Paola Schutz, Virginia Bove, Fiorella Villano, Cecilia Beltran, Javiera Donoso, Hernán López-Vidal, Macarena Alejandra Roa Salinas, Pablo Soto, Paola Ochoa, Patricio Duarte, Guillermina Remaggi, Ariel Corzo, Claudia Shanley, Sergio Lopresti, Sergio Orlando, Verónica Verri, Luis Darío Quiroga, Dorotea Fantl, Jhoanna Ramirez, Alejandro Ospina-Idárraga, Henry Idrobo, Guillermo Quintero, Rigoberto Gomez, Omar Cantú-Martínez, David Gomez-Almaguer, Guillermo J. Ruiz-Arguelles, Kenny Mauricio Galvez-Cárdenas, Luis Antonio Salazar, Isabella Novoa-Caicedo, María Cynthia Fuentes-Lacouture, Paola Spirko, María Isabel Arbeláez, Mario Pereira, Jaime Valdes, Jule Vasquez, Alana von Glasenapp, Eloísa Riva

**Affiliations:** ^1^ Instituto Nacional de Cancerología de Colombia, Bogotá, Colombia; ^2^ Hospital Militar Central, Bogotá, Colombia; ^3^ Departamento de Hematología, Hospital Del Salvador, Santiago, Chile; ^4^ Hospital Italiano de Buenos Aires, Buenos Aires, Argentina; ^5^ Asociación Española, Montevideo, Uruguay; ^6^ CASMU, Montevideo, Uruguay; ^7^ Hospital de Temuco, Temuco, Chile; ^8^ Hospital Sótero del Río, Santiago, Chile; ^9^ Hospital Barros Luco, Santiago, Chile; ^10^ Hospital Dr. Eduardo Schütz Schroeder, Puerto Montt, Chile; ^11^ Instituto Alexander Fleming, Munro, Argentina; ^12^ CEMIC University Hospital, Buenos Aires, Argentina; ^13^ Fundaleu, Buenos Aires, Argentina; ^14^ Hospital de Clínicas, Buenos Aires, Argentina; ^15^ Hospital Británico, Buenos Aires, Argentina; ^16^ Hospital Posadas, El Palomar, Argentina; ^17^ Hospital Rossi, La Plata, Argentina; ^18^ IDIM, Buenos Aires, Argentina; ^19^ Hospital Churruca, Buenos Aires, Argentina; ^20^ Hospital IESS, Guayaquil, Ecuador; ^21^ Hospital Universitario Del Valle Evaristo García E.S.E, Cali, Colombia; ^22^ Hospital Universitario Fundación Santa Fe de Bogotá, Bogotá, Colombia; ^23^ Instituto Catalán de Oncología, Cali, Colombia; ^24^ Hospital Universitario, Universidad Autónoma de Nuevo León, Monterrey, México; ^25^ Hospital Universitario Dr. José Eleuterio González, Monterrey, México; ^26^ Centro De Hematología Y Medicina Interna, Puebla, México; ^27^ Hospital Pablo Tobón Uribe, Medellín, Colombia; ^28^ Clínica FOSCAL, Bucaramanga, Colombia; ^29^ Facultad de Medicina, Universidad El Bosque. Bogotá, Colombia; ^30^ Instituto Nacional De Enfermedades Neoplásicas, Lima, Perú; ^31^ ReVita, Centro Oncológico Integral, Asunción, Paraguay; ^32^ Hospital de Clínicas, Montevideo, Uruguay

## Abstract

**PURPOSE:**

Multiple myeloma (MM) is a highly heterogeneous, incurable disease most frequently diagnosed in the elderly. Therefore, data on clinical characteristics and outcomes in the very young population are scarce.

**PATIENTS AND METHODS:**

We analyzed clinical characteristics, response to treatment, and survival in 103 patients with newly diagnosed MM age 40 years or younger compared with 256 patients age 41-50 years and 957 patients age 51 years or older.

**RESULTS:**

There were no statistical differences in sex, isotype, International Scoring System, renal involvement, hypercalcemia, anemia, dialysis, bony lesions, extramedullary disease, and lactate dehydrogenase (LDH). The most used regimen in young patients was cyclophosphamide, bortezomib, dexamethasone, followed by cyclophosphamide, thalidomide, dexamethasone and bortezomib, thalidomide, dexamethasone. Of the patients age 40 years or younger, only 53% received autologous stem-cell transplant (ASCT) and 71.1% received maintenance. There were no differences in overall survival (OS) in the three patient cohorts. In the multivariate analysis, only high LDH, high cytogenetic risk, and ASCT were statistically associated with survival.

**CONCLUSION:**

In conclusion, younger patients with MM in Latin America have similar clinical characteristics, responses, and OS compared with the elderly.

## Teaser

Outcomes of Multiple myeloma patients aged 40 years and younger in LATAM

## INTRODUCTION

Multiple myeloma (MM) is a heterogeneous disease most often diagnosed in people older than 65 years, and it is very rare in people younger than 40 years. Data on clinical characteristics, response to treatment, and survival in this younger population are scarce. Unfortunately, even in this young group, it remains incurable despite the availability of modern treatments.^1,2^ The first retrospective series from the Mayo Clinic showed that patients younger than 40 years represent <3% of all patients with MM, and MM is extremely infrequent under 30 years. The MM in the young population is more frequent in men; it has a higher frequency of light chain MM, especially in those younger than 30 years, who also show significant bone involvement with extramedullary dissemination, a low monoclonal protein component, and few plasma cells in the bone marrow.^3,4^ Better survival has been described in this population group of patients with MM, attributed to better tolerance to chemotherapy and because within the studied cohorts, there is a percentage of patients with lower tumor burden and the absence of renal involvement whose survival could exceed 8 years, even before new therapies were introduced.^3,4^ The average number of years of life lost in patients with MM is more significant than in many other cancers and could exceed 30 years in patients younger than 40 years of age.^5^ Therefore, the younger population with MM would require a different therapeutic approach in the era of new therapies. This article presents the demographic and clinical characteristics of response to treatment and survival of our population younger than 40 years, specifically in Latin American patients belonging to the Grupo de Estudio Latinoamericano de Mieloma Múltiple (GELAMM) database.

**CONTEXT**

**Key Objective**

The key objective of this manuscript is to highlight the behavior of young patients with multiple myeloma (MM) in Latin America, in order to define if there is any difference in the clinical behavior of these patients and if they deserve a different approach.

**Knowledge Generated**

The knowledge generated by this manuscript emphasizes that these patients could have a similar behavior to the rest of the population; however, it also highlights the difficulty in accessing consolidation therapies such as hematopoietic stem cell transplantation in Latin America, thus, generating a urgent need to correct the needs in access to these therapies. It also highlights their impact on the evolution of the disease over time.

**Relevance**

To our knowledge, this is one of the few studies that focus on young patients with MM in order to evaluate their clinical behavior. With these results we can impact the necessary consolidation therapies for young patients with the disease, in whom the requirement for innovative treatments is accentuated.

## PATIENTS AND METHODS

We analyzed newly diagnosed multiple myeloma (NDMM) patients younger than 40 years who received treatment for MM using international myeloma working group (IMWG) criteria for diagnosis in six Latin American countries between 2010 and 2018 from the GELAMM database. Demographics and disease features were analyzed using descriptive statistics. We included the variables of age, sex, isotype, International Scoring System, renal involvement (creatinine ≥2), hypercalcemia (calcium ≥10), anemia (hemoglobin ≤10), dialysis, bony lesions, extramedullary disease, high lactate dehydrogenase (LDH), high-risk cytogenetics (CG; t4;14, t14;16, del 17p), treatment received, response to treatment (pre- and post-transplant), maintenance therapy, and overall survival (OS). Differences in clinical presentation between groups were compared using the chi-square test and standardized mean differences (SMDs). Estimates of OS within age groups were produced using the Kaplan-Meier method and were compared using the log-rank test among the age population. Multivariate analysis was performed using Cox regression analysis. Progression-free survival (PFS) was not analyzed because of the missing data.

Patients with smoldering (asymptomatic) myeloma, amyloidosis, and monoclonal immunoglobulin M–related disorders were not included. All analyses were performed using the statistical package SPSS version 25 (Armonk, NY).

## RESULTS

One thousand three hundred sixteen patients were included in this analysis. Of the total database, 7.8% were patients younger than 40 years. The median age of all combined cohorts was 53.81 years, whereas the median age of younger patients was 35.14 years, and 64.1% were male. The presenting features of patients age 40 years and younger compared with other age populations are shown in Table 1.

**TABLE 1:**
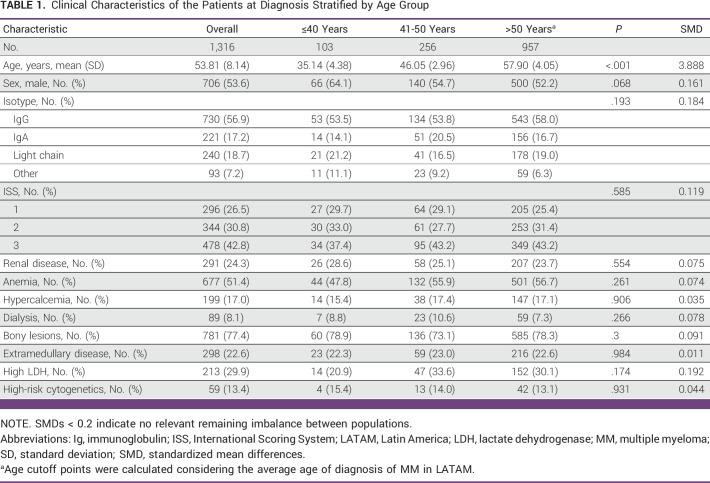
Clinical Characteristics of the Patients at Diagnosis Stratified by Age Group

Regarding treatment, cyclophosphamide, bortezomib, dexamethasone (CyBorD) was the most used regimen, followed by cyclophosphamide, thalidomide, dexamethasone (CTD) and bortezomib, thalidomide, dexamethasone (VTD) in the younger population; lenalidomide was only prescribed as a frontline regimen in four patients, 3.9%, which was similar in the older population (Table 2). Fifty-five patients (53%) in this younger population received high-dose therapy and autologous stem-cell transplantation (ASCT), and only 45% received maintenance therapy without bone marrow transplant.

**TABLE 2:**
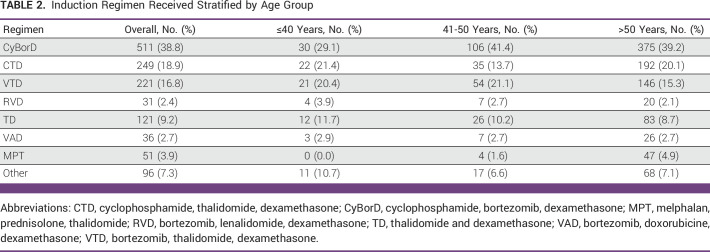
Induction Regimen Received Stratified by Age Group

The type of maintenance received was thalidomide in 47.7%, lenalidomide in 45.3%, bortezomib in 4.5%, and others in 2.3%.

The overall response rate in ASCT was 78.8% (Table 3). For patients younger than 40 years, it was 78.6%; in patients age 41-50 years, it was 78.6%; and it was 80.3% in patients older than 50 years.

**TABLE 3:**
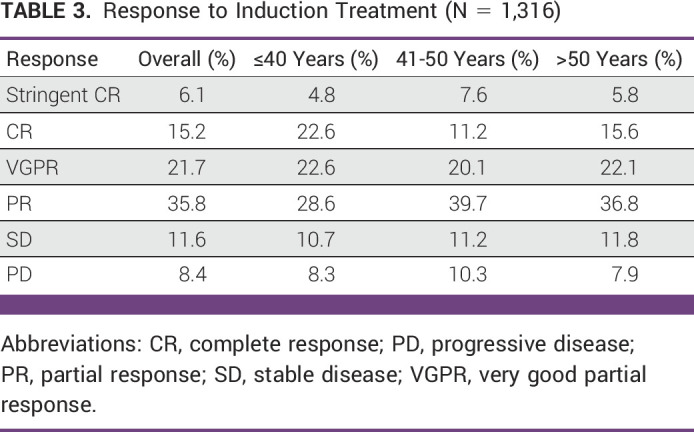
Response to Induction Treatment (N = 1,316)

The overall response rate post-ASCT was 93% in patients younger than 40 years, 94.2% in patients age 41-50 years, and 94.4% in those older than 50 years, indicating that ASCT deepens responses achieved at the induction time. An interesting finding is that patients age 40 years or younger tend to achieve more complete responses after bone marrow transplant than patients in the group of 41 to 50 years and older than 51 years (34.9% *v* 24.6%, *v* 28.7%, respectively; Table 4).

**TABLE 4:**
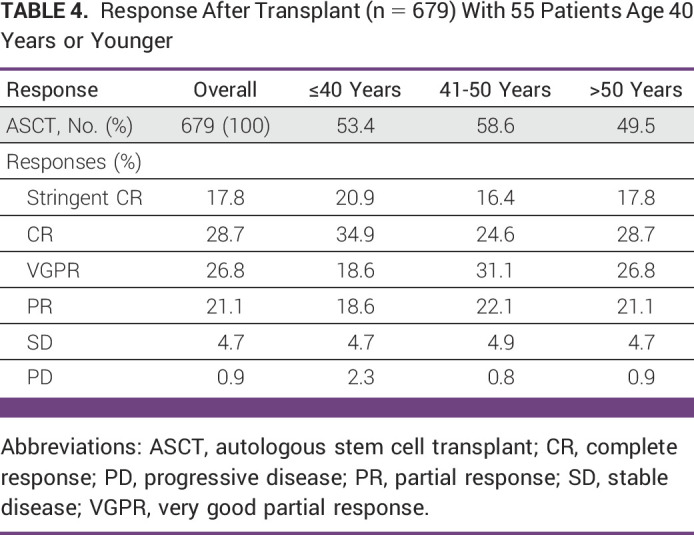
Response After Transplant (n = 679) With 55 Patients Age 40 Years or Younger

**FIG 1:**
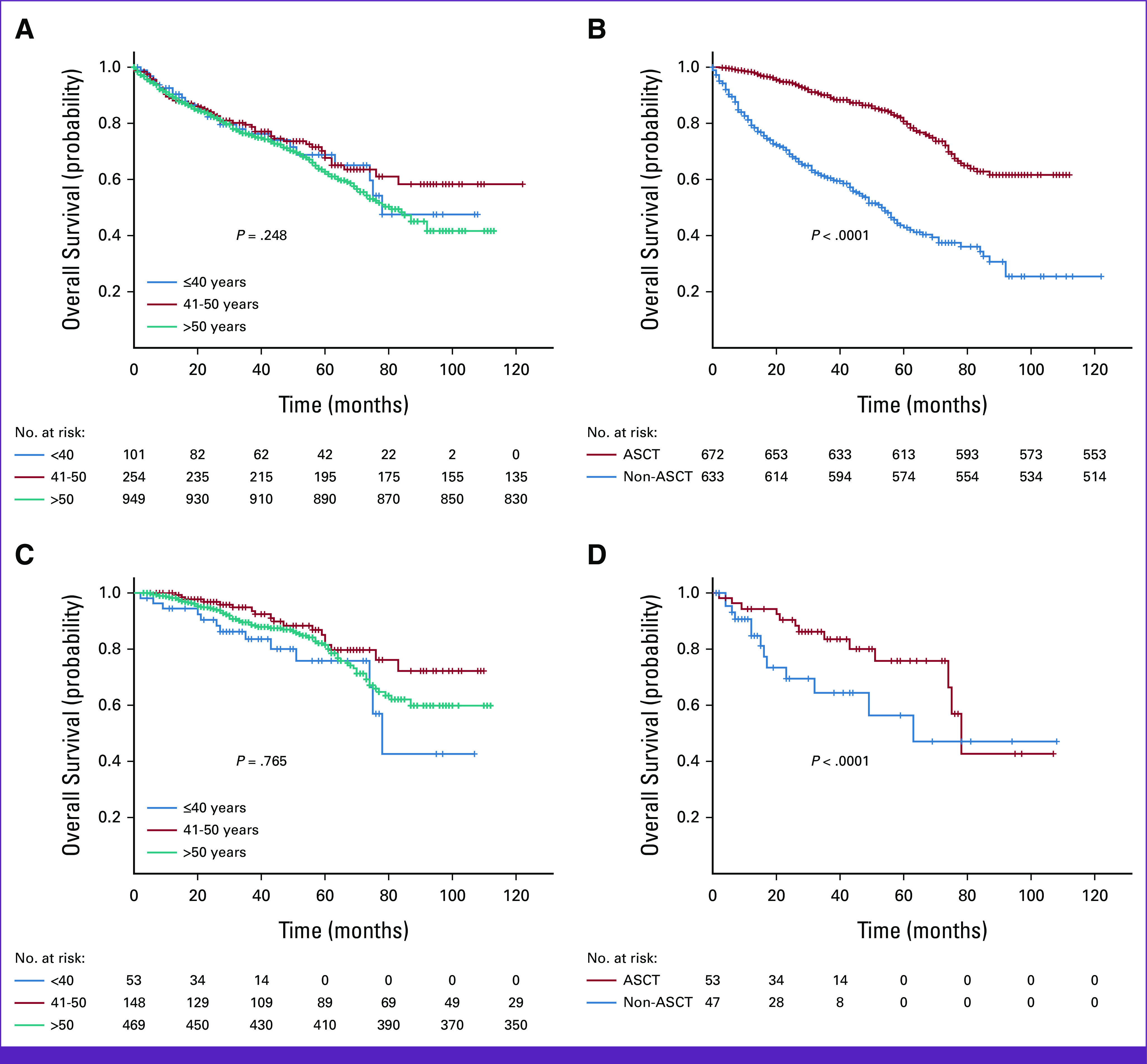
Survival analyses. (A) Overall survival for the entire cohort. (B) Transplanted (red line) *v* nontransplanted (blue line) patients in the entire cohort. (C) The entire cohort of patients undergoing ASCT. (D) Only patients age 40 years or younger ASCT (red line) versus non-ASCT (blue line). ASCT, autologous stem-cell transplant.

With a median follow-up of 32 months (range, 1-113), the median OS for the entire cohort was 85 months (95% CI, 81.413 to 88.587), with no statistically significant differences for the three groups (*P* = .248). The median OS for the entire cohort that did not proceed with ASCT was 53 months (95% CI, 46.8 to 59.1), whereas for the cohort that did proceed with ASCT, the median survival was not achieved (*P* < .0001; Fig 1B). No statistically significant differences across the three groups of patients taken to ASCT were observed (*P* = .940; Fig 1C). In the group 40 years or younger, the median OS in the nontransplanted group was 63 months (95% CI, 57.67 to 68.33) and 78 months (95% CI, 53.444 to 102.56) in the patients taken to ASCT (*P* < .001).

In the multivariate analysis (Table 5), the factors that independently correlated with better survival were not high LDH, not high risk, and having proceeded with ASCT.

**TABLE 5:**
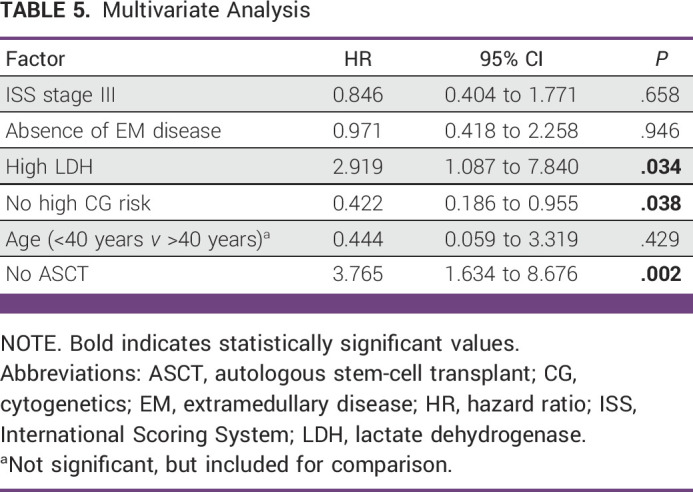
Multivariate Analysis

## DISCUSSION

This retrospective international multicenter cohort study focuses on the outcomes of patients age 40 years or younger with MM in our region. MM remains an incurable disease in most patients although its prognosis has improved in the past two decades.^1,6^ Several factors explain this improvement in survival, including the use of triple combinations, post-transplant maintenance therapy in eligible patients or continuous therapy in ineligible patients, the possibility of achieving minimal negative residual disease status, and the use of novel therapies including immuno-effector cell therapy.^7-9^ The median age of patients at diagnosis of MM is 65 years. The population younger than 50 years is 13%, the population younger than 40 years is only 2.2%, and the population younger than 30 years is up to 0.3%.^3,4^ Our study shows that 7.8% are 30 years old or younger, indicating that Latin America's MM population tends to be younger. However, red flags for back pain, renal failure, or anemia are more easily detectable in the young population, and we add this to the fact that MM diagnosis in the older population is sometimes difficult, even more in Latin America where access to medical specialists can be limited, these numbers must be taken cautiously.

Although several studies have shown that the clinical presentation of younger patients may differ from that of older patients, we found that the clinical characteristics that define the disease are the same in both age groups.^2-4^ The treatment of MM in our study was quite heterogeneous; however, the main treatments are VTD and CyBorD, which reflects Latin American clinical practice, and access to lenalidomide, the standard of care in other latitudes, was only offered to very few patients.^10,11^

Similarly, consolidation with high doses of chemotherapy and autologous cell salvage was offered only to 53% of patients, and maintenance to less than half, which denotes the marked disparities with other latitudes.^12,13^ The response to induction treatment was similar in the three cohorts, and the post-transplant response in patients who achieved transplantation tended to be slightly better in patients younger than 40 years. The OS of our entire cohort is 85 months, which is lower than that reported for populations treated with similar schemes.^14^ The prognosis of patients younger than 40 years has been reported to be better than that in the older population when they present with favorable risk factors, which did not occur in our study.^3,4^ This could also be explained by the high number of patients who were not taken for transplantation, thus reflecting an unmet need for treatment in Latin America. Despite the prognosis seeming to be better in other studies, the years of life lost clearly in this population are many, reaching up to more than 30 years.^5^ The preceding shows that the therapeutic approach of these patients must be different, considering the possible better tolerance to more intensive treatments where, without a doubt, the new therapeutic options will play a fundamental role.^15,16^

Among the limitations of our study are the low number of patients, the inability to measure PFS to the main treatments used, and the nonregistration of data on toxicity.

In conclusion, in this Latin American multicenter study, we found that the young population with MM has similar presentation characteristics to elderly patients. A significant amount of information is lost regarding the risk characterization, especially regarding CG. Regarding treatment, less than half of the patients achieve a very good partial response or better. It is striking that more than a third of these young patients did not have access to high doses of chemotherapy and bone marrow transplantation. Maintenance therapy is offered to less than half of the patients. The median OS is lower than that in other series of patients younger than 40 years, even than in the elderly cohorts. Prospective multicentric studies are required to elucidate the behavior of the disease in this group of patients.

## References

[1] Ravi P Kumar SK Cerhan JR et al Defining cure in multiple myeloma: A comparative study of outcomes of young individuals with myeloma and curable hematologic malignancies Blood Cancer J 8 26 2018 29531285 10.1038/s41408-018-0065-8 PMC5849889

[2] Caulier A Roussel M Morel P et al Epidemiological landscape of young patients with multiple myeloma diagnosed before 40 years of age: The French experience Blood 138 2686 2695 2021 34479366 10.1182/blood.2021011285

[3] Bladé J Kyle RA Greipp PR Presenting features and prognosis in 72 patients with multiple myeloma who were younger than 40 years Br J Haematol 93 345 351 1996 8639427 10.1046/j.1365-2141.1996.5191061.x

[4] Ludwig H Durie BG Bolejack V et al Myeloma in patients younger than age 50 years presents with more favorable features and shows better survival: An analysis of 10 549 patients from the International Myeloma Working Group Blood 111 4039 4047 2008 18268097 10.1182/blood-2007-03-081018 PMC4259800

[5] Ludwig H Bolejack V Crowley J et al Survival and years of life lost in different age cohorts of patients with multiple myeloma J Clin Oncol 28 1599 1605 2010 20177027 10.1200/JCO.2009.25.2114

[6] Shimazu Y Mizuno S Fuchida SI et al Improved survival of multiple myeloma patients treated with autologous transplantation in the modern era of new medicine Cancer Sci 112 5034 5045 2021 34644446 10.1111/cas.15163 PMC8645729

[7] Kastritis E Terpos E Dimopoulos MA How I treat relapsed multiple myeloma Blood 139 2904 2917 2022 35007326 10.1182/blood.2020008734

[8] Mikkilineni L Kochenderfer JN CAR T cell therapies for patients with multiple myeloma Nat Rev Clin Oncol 18 71 84 2021 32978608 10.1038/s41571-020-0427-6

[9] Kazandjian D Multiple myeloma epidemiology and survival: A unique malignancy Semin Oncol 43 676 681 2016 28061985 10.1053/j.seminoncol.2016.11.004 PMC5283695

[10] de Moraes Hungria VT Martínez-Baños DM Peñafiel CR et al Multiple myeloma treatment patterns and clinical outcomes in the Latin America Haemato-Oncology (HOLA) Observational Study, 2008-2016 Br J Haematol 188 383 393 2020 31392724 10.1111/bjh.16124 PMC7003731

[11] Peña C Riva E Schutz N et al Different outcomes for transplant-eligible newly diagnosed multiple myeloma patients in Latin America according to the public versus private management: A GELAMM study Leuk Lymphoma 61 3112 3119 2020 32844699 10.1080/10428194.2020.1804558

[12] Ludwig H Novis Durie S Meckl A et al Multiple myeloma incidence and mortality around the globe; interrelations between health access and quality, economic resources, and patient empowerment Oncologist 25 e1406 e1413 2020 32335971 10.1634/theoncologist.2020-0141 PMC7485361

[13] Riva E Schütz N Peña C et al Significant differences in access to tests and treatments for multiple myeloma between public and private systems in Latin America. Results of a Latin American survey. GELAMM (Grupo de Estudio Latino Americano de Mieloma Múltiple) Ann Hematol 99 1025 1030 2020 32157420 10.1007/s00277-020-03983-x

[14] Reeder CB Reece DE Kukreti V et al Cyclophosphamide, bortezomib and dexamethasone induction for newly diagnosed multiple myeloma: High response rates in a phase II clinical trial Leukemia 23 1337 1341 2009 19225538 10.1038/leu.2009.26 PMC2711213

[15] Rodríguez-Otero P Prósper F Alfonso A et al CAR T-cells in multiple myeloma are ready for prime time J Clin Med 9 3577 2020 33172026 10.3390/jcm9113577 PMC7694626

[16] Moore DC Oxencis CJ Shank BR New and emerging pharmacotherapies for the management of multiple myeloma Am J Health Syst Pharm 79 1137 1145 2022 35333922 10.1093/ajhp/zxac091

